# A Microfluidic Device to Realize Electrochemically Controlled SERS Detection in HPLC

**DOI:** 10.1021/acs.analchem.5c02232

**Published:** 2025-06-23

**Authors:** Maximilian E. Blaha, Julius Schwieger, Rico Warias, Anish Das, Matthias Polack, Detlev Belder

**Affiliations:** Institute for Analytical Chemistry, 9180 Leipzig University, Linnéstraße 3, Leipzig 04103, Germany

## Abstract

Surface-enhanced Raman spectroscopy (SERS) is a powerful technique for vibrational spectroscopy, but analyzing mixtures in solution remains challenging due to spectral overlap. Integrating SERS with a separation method, such as high-performance liquid chromatography (HPLC), offers a promising solution. However, online coupling has been limited by the compatibility issues between the SERS process and flow-based systems, which can result in either irreversible analyte adsorption on the SERS substrate or insufficient interaction. This can lead to signal carry-over or low sensitivity. In this study, we present the first HPLC-compatible, pressure-stable SERS flow cell designed for real-time analysis under continuous flow. Fabricated entirely from glass using selective laser etching, the monolithic flow cell incorporates a silver-based SERS substrate and a counter electrode, enabling online electrochemical SERS (EC-SERS) experiments. Electrochemical control facilitates on-demand substrate activation, thereby enhancing signal intensity, extending substrate lifetime, and eliminating memory effects. This approach broadens the range of detectable analytes, including those that are traditionally difficult to detect using passive SERS. We demonstrate the performance of the system through HPLC-SERS analyses of model dyes (e.g., crystal violet, malachite green, and rhodamine) and pharmaceutical compounds (e.g., cyanocobalamin and folic acid). This innovation introduces a novel SERS-based HPLC detection method, supporting the seamless integration of SERS into high-throughput analytical workflows.

## Graphical abstract

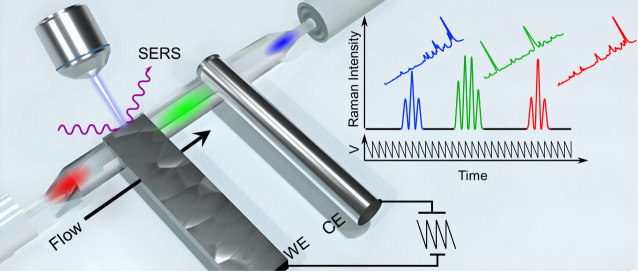

## Introduction

Surface Enhanced Raman Spectroscopy (SERS) is an analytical technique that amplifies the inherently weak Raman signals of molecules adsorbed onto roughened, plasmonically active metal surfaces, commonly referred to as SERS substrates.^1–3^ This enhancement can be as high as 10^1 1^, theoretically enabling the detection of diluted molecules^4,5^ down to a single molecular level, providing detailed vibrational spectra for molecular identification.^6–8^ Thus, it is no surprise that SERS has been used for multiple analytical applications in pharmaceutics, food chemistry, bioanalytics and trace nanoplastic detection.^9–12^ The objective of our work is to integrate this promising technology with high-performance liquid chromatography (HPLC).

The combination of HPLC with SERS is of great interest to the scientific community. However, the current approaches to coupling HPLC with SERS either involve using highly SERS-active model compounds, such as dyes or sulfur-containing molecules, at high concentrations,^13,14^ or are performed offline after separation.^15–19^ We aim to broaden the applicability of online SERS by incorporating electrochemical techniques, leading to the development of electrochemical SERS (EC-SERS)^20–23^ sensors for HPLC. The inclusion of electrochemical methods and the resulting spectroelectrochemistry can potentially expand SERS with specific features and overcome some of its limitations.

Despite its promising features, SERS faces several significant drawbacks that limit its widespread use,^24–28^ especially its compatibility with online separation methods such as HPLC.^25^ The short contact time between the analyte-containing solution and the SERS substrate may lead to poor adsorption of some target molecules, rendering them undetectable. To overcome low signal intensities caused by poor adsorption, current methods involve incubating the analyte-containing solution on the SERS substrate or allowing it to dry to maximize surface loading.^29–31^ However, both approaches are either incompatible with online HPLC or significantly increase the complexity of the system. Therefore, integrating electrochemical methods facilitates the rapid adsorption of analytes onto the surface, thereby enhancing signal intensity.^20,32–36^ This approach also renders otherwise undetectable species observable, as demonstrated in this work.

Another issue associated with commonly used silver-based SERS-substrates is that they slowly corrode over time. The formation of a thin layer of sulfides or silver oxide hinders the plasmonic activity, thereby reducing the effectiveness of the substrate. Electrochemical techniques can activate the SERS substrate^37^ and create a roughened surface,^38–40^ thus allowing for more significant and prolonged use of a SERS substrate inside a flow cell.

Another advantage of electrochemical methods is their ability to alter the adsorbed substances, either by changing the bonding angle or by inducing oxidation or reduction of the compound.^38,39,41,42^ This can result in a modified or more intense spectrum of the target molecule, providing additional information. Altered spectra can contribute to higher signal intensities, making substances detectable in the first place, while also providing a specific identification feature.

Another challenge is the “memory effect,” which refers to the prolonged adsorption of target molecules on the SERS substrate.^43–45^ In the context of the intended use of SERS as an HPLC detector, the memory effect can lead to carryover issues,^46^ resulting in overlapping signals of successively eluting species. Common strategies to overcome the memory effect in SERS include using disposable substrates, stripping adsorbed chemicals through harsh conditions such as corrosive agents,^47,48^ heavily flushing with solvents,^49,50^ or UV radiation,^51^ and preventing undesired adsorption by applying thin polymer coatings.^52^ The electrochemical alteration of either the SERS-substrate or the analyte molecule on the surface facilitates reductive^33,34,37,53,54^ (or oxidative^44,55^ analyte stripping to mitigate the memory effect. Depending on the applied potential, it can also enhance analyte adsorption and increase signal intensities.^32–36,56^

In this work, we present a pressure-stable flow cell made from fused silica that integrates a broadband SERS substrate with a counter electrode. The microfluidic device was manufactured using selective laser-induced etching (SLE) techniques. We showcase its application in online methods, utilizing it as a SERS detection cell in HPLC. Our EC-SERS approach effectively activates the SERS substrate, addresses the memory effect, enhances SERS intensities, and enables the detection of analytes that would otherwise be invisible by SERS. We provide a comprehensive guide for manufacturing and handling the sensor to optimize experimental results. The sensor’s efficacy is first illustrated through HPLC separation of model compounds (such as crystal violet, malachite green, and rhodamine B), and further validated with the analysis of Vitamin B12 (cyanocobalamin) and Vitamin B9 (folic acid).

## Materials and Methods

### Chemicals

The silver wires (diameter 0.25 mm, 99.9%) were purchased from Sigma-Aldrich, Steinheim, Germany. Platinum wires (diameter 0.25 mm, 99.9%) were purchased from Alfa Aesar GmbH, Karlsruhe, Germany. Analytes were purchased as follows: Crystal violet (Sigma-Aldrich), Malachite green (Kallies Feinchemie KG, Sebnitz, Germany), Rhodamine B (Sigma-Aldrich), Riboflavin (Sigma-Aldrich), Cyanocobalamin (Caesar and Loretz GmbH, Hilden, Germany), Folic acid (BLD-Pharm, Reinbek, Germany). Electrolytes: NaOAc (Merck KGaA, Darmstadt, Germany), Bu_4_NOAc (BLD-Pharm), Acetic acid (Carl Roth GmbH & Co. KG, Karlsruhe, Germany). Reagents were purchased as follows: NH_3_ 35% (Fisher), HNO_3_ 65% (Merck), MeOH (HiPerSolv Chromanorm, VWR, Darmstadt, Germany), MeCN (VWR, Darmstadt, Germany), H_2_O (deionized), Dioctyl sulfosuccinate sodium salt (Sigma-Aldrich), KOH 8 M (Carl Roth).

### SERS-Substrate Manufacturing

Similar to our previous study,^53^ we utilized an etched silver wire adapted from the method developed by Wijesuriya, Burugapalli et al.^57^ The silver wire was flattened using a mechanical press (PO10H; Paul Otto Weber; Germany) for 120 min at a pressure of 30 kN. Afterward, the wire was polished (Lapping Film 261X, 3M, USA), sanded down to the required shape required for insertion into the microfluidic device, and ultrasonicated in methanol (MeOH) for 3 min. The etching process involved immersing the flattened wire in 35% ammonia (NH_3_) for 30 s, followed by 6 M nitric acid (HNO_3_). The wire was etched until it began to lose its gloss (approximately 10 s).

### Microfluidic Device Design and Manufacturing

The EC-SERS flow cell was manufactured using selective laser-induced etching. The procedure was adapted from previous work as follows:^58^ The device was designed using Autodesk Inventor Professional 2024 (San Rafael, CA, USA), taking into account the etch rate of pristine fused silica material (1 μm/h) for dimensional accuracy. The design included inserts for fused silica capillary tubings (outer diameter: 360 μm; inner diameter: 100 μm), the platinum counter electrode (diameter: 250 μm), and the etched SERS substrate as the working electrode. The CAD file was converted to the. step file format and further processed with Alphacam 2017 R2 (Vero Software GmbH, Neu-Isenburg, Germany) CAM software. The laser structuring was performed on a 4″ fused silica wafer (Siegert Wafer GmbH, Aachen, Germany) with a thickness of 1 mm and an SLE device (FEMTOprint aHead P2, FEMTOprint SA, Muzzano, Switzerland). For structuring, a 20 × lens (LHM-20X-1064, NA = 0.40) and a pulsed IR-laser (λ = 1030 nm; 400 fs) were used. Process parameters were as follows: laser energy: 230 nJ, repetition rate: 1 MHz, feed rate: 15.83 mm s^–1^, layer distance horizontal: 2 μm, layer distance vertical: 7 μm. The resulting substrate was etched for 21 h in 8 M KOH with 0.02 wt % dioctyl sulfosuccinate sodium salt at 85 °C in a pulsed ultrasonic bath (15 min period duration; 2 min duty).

For the assembly, the Pt wire, the etched Ag wire (SERS substrate), and fused silica capillaries were glued into the device inserts using ClearWeld (J-B Weld, TX, USA). The assembled microfluidic EC-SERS device was left for curing overnight.

### Raman Setup

For Raman signal acquisition, a system similar to that used in previous works was used.^53^ The chip was placed on an IX71 inverted microscope (Olympus, Japan) equipped with a LUCPlanFl 40× objective lens (NA 0.6; Olympus, Japan). The optical setup is based on a confocal modular Raman measurement system (S&I Spectroscopy & Imaging GmbH, Germany). A 473 nm Cobolt Blues 50 mW laser (Cobolt AB, Sweden) was used as the excitation source. The excitation light was directed through a neutral density filter wheel and a dichroic mirror (DC) onto the SERS substrate. The scattered light was collected by the same lens, passed through an edge filter (EF), and was then directed into a spectrograph/monochromator (Acton SP2750, Princeton Instruments, USA) with a 150 μm entrance slit and a grating of 600 lines/mm. Detection was carried out using a Peltier-cooled 1600 × 200 CCD camera (ProEM, Princeton Instruments, USA). The laser power used for each experiment is specified in the corresponding description. The CCD camera was read out by a PC running VistaControl V4.8.3 (S&I Spectroscopy & Imaging GmbH). Simplified schematics of the Raman setup are provided in Figure 1.

**Figure 1:**
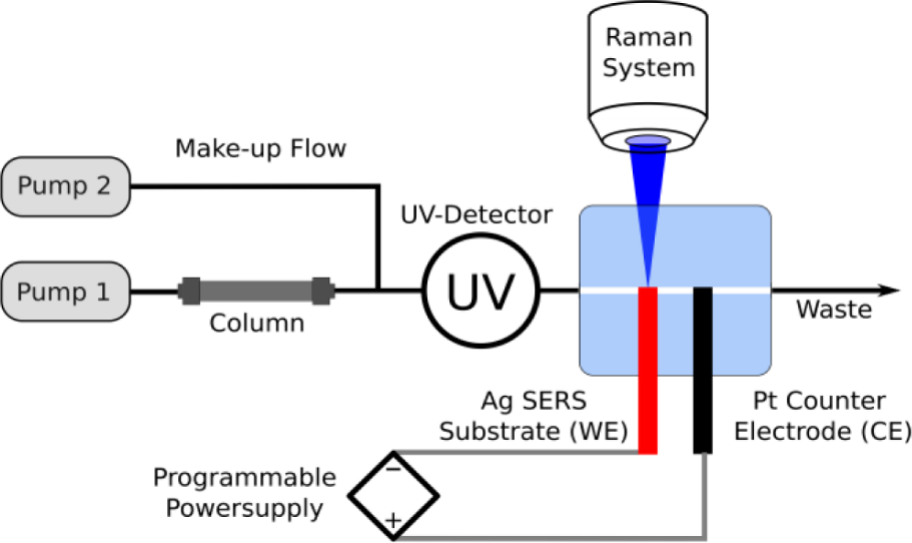
Experimental setup.

### Fluidic Setup for Calibration and Preparation

Calibration experiments are necessary to verify the SERS substrate’s performance and to optimize the focus of the optical setup for maximum intensity. We used a syringe pump (Pump II, Harvard Apparatus, Massachusetts, USA) equipped with a 2.5 mL glass syringe (1000 series with Luer tip, Hamilton, Giarmata, Timis County, Romania). 10 μM Crystal violet was dissolved in 30:70 MeOH:H_2_O containing 50 mM NaAc/HAc buffer with a pH of 4.8. The solution was guided through a syringe filter (4 mm Nylon, 0.22 μm, BGB, Lörrach, Germany) using standard PEEK or PTFE tubing and adapters directly onto the sensor. To activate the SERS substrate, a reductive potential of −8 V was applied for 10 min while the crystal violet solution was pumped with a flow rate of 30 μL/min through the EC-SERS-HPLC-Sensor. Afterward, the electrical circuit was interrupted. The focus spot of the laser was adjusted to maximize intensity.

### HPLC and Fluidics Setup for EC-SERS-Coupling

A 1260 Infinity pump (Agilent) was used for HPLC separations coupled with EC-SERS detection. A 7725i hexaport injector valve (Rheodyne, IDEX Health & Science LLC.) with needle port and electrical switch was used for sampling. Used sample volumes were 100 μL for Raman model dye separations and 20 μL for vitamin separations. After the hexaport valve, the injected analytes reached the column (Eclipse Plus C18, Agilent). A makeup liquid was added after the column to add supporting electrolytes and modify the eluent composition to support EC-SERS measurements. For the EC-SERS/HPLC analysis of the Raman model dyes, pure water containing 50 mM NaAc/HAc buffer was added at a flow rate of 600 μL/min using an additional 1260 Infinity pump. For EC-SERS/HPLC analysis of vitamins, a makeup flow containing 250 mM Bu_4_NOAc was added with a flow rate of 100 μL/min using a Dossier/HPLC-Pump 3350 (BISCHOFF Analysentechnik u. -geräte GmbH, Leonberg, Germany). For all connections, standard PEEK-tubings (OD: 1/16″, ID: 0.25 – 0.13 mm, BGB, Lörrach, Germany) and adapters were used. In addition to the newly developed EC-SERS detection module, a UV detector (Spectra 100, Spectra Physics, CA, USA) was also connected in series to monitor the HPLC separations. A schematic of the experimental setup is shown in Figure 1.

### Electronics, Data Acquisition, and Synchronization

A program was developed using LabVIEW 2017 (National Instruments, USA), which utilized an I/O Device (USB-6001, National Instruments, USA) to synchronize all connected electronics by reading and writing trigger pulses to the relevant systems. The program is capable of reading data from the UV detector and serves as a programmable power supply for the sensor. For synchronization, the program waits for the opening of the hexaport injector valve. Upon the valve’s activation, the CCD camera of the Raman system is triggered and a preset voltage program starts, which controls the EC-SERS process. Simultaneously, it begins reading data from the UV detector while monitoring the current and potential of the applied voltage program. The electronics behind the programmable power supply are adapted from the current literature of self-made potentiostats^59,60^ and are used in a two-electrode configuration. It incorporates OpAmps (LM741, Texas Instruments, TX, USA) to amplify the signal from the I/O-device to measure the current. We used a precision multimeter (DMM6500, Keithley, Ohio, USA) to read the current from this monitor. The potential was monitored using the I/O–Device. A standard laboratory power supply (DC Dual Power Supply 6145, PeakTech, Ahrensburg, Germany) provided power to the circuit. A more detailed description of the electronics is available in the Supporting Information.

## Results and Discussion

Our work aims to establish surface-enhanced Raman spectroscopy (SERS) as a real-time and online detection method for high-performance liquid chromatography (HPLC). To be used as a detection method in HPLC, SERS must be able to recognize compounds that are eluted in rapid succession in real-time and without interference. The combination of SERS with HPLC is challenging due to the nature of SERS, which requires a strong interaction between the analyte and the SERS substrate; however, this effect can lead to carryover and thus impair the detection after HPLC separation. Furthermore, the high flow rates typically used in HPLC result in very short residence times of the analytes in the detection cell. The resulting short contact times of the analyte with the SERS substrate can lead to low signal intensities and the undetectability of certain compounds. To overcome these limitations, we propose implementing spectroelectrochemistry to enhance signal intensity, facilitate substrate regeneration and activation, and mitigate memory effects. This requires the development of a system that includes a programmable power source, a Raman spectrometer, and a pressure-stable flow cell with an electrically contactable SERS substrate and a counter electrode.

When designing a detection flow cell for high-performance separation methods such as HPLC, one key aspect is that the detection cell must be pressure and solvent-resistant. Furthermore, it must avoid peak broadening. Therefore, the detection cell must be smaller than the peak volumes eluting from the column, and the connection technology must be designed to minimize dead volumes. In our earlier studies on fundamental aspects of EC-SERS detection in flow, we used a simple electrochemical flow cell with laminated foils that does not fulfill these requirements.^53,61^ First, it contains far too large a volume of 3 μL, the simple connection technique is prone to peak spreading and carry over and the plastic films and adhesives are neither solvent nor pressure stable.

### The EC-SERS/HPLC Flow Cell

We have utilized selective laser etching to develop a miniaturized spectrochemical flow cell suitable for high-performance liquid chromatography (HPLC). This technology enables the generation of complex three-dimensional structures in monolithic fused-silica material, characterized by excellent mechanical and chemical stability, as well as high optical quality. This technique also enables the production of round bushings for the dead volume-free insertion of connection capillaries^62,63^ and the introduction of electrodes for spectroelectrochemical processes.

This chip-like microfluidic device, which contains a wet-etched Ag-based SERS substrate, a Pt counter electrode, and fluidic connections, is schematically illustrated in Figure 1, along with other components in the measurement setup. The entire setup includes a modular HPLC instrument, a UV detector, the microfluidic flow cell, a Raman microscope, a programmable potentiostat and a makeup flow dosing system to adopt the solvent for spectroelectrochemical measurements without affecting the HPLC separation. A detailed description of the setup is provided in SI Chapter 1.

A photo of the assembled device, a light microscopic image of the detection cell and a schematic view are shown in Figure 2. Fused silica capillaries with an outer diameter of 360 μm and an inner diameter of 100 μm were inserted and used as fluidic connections. As a SERS substrate (working electrode, WE), we used the wet-etched Ag-based SERS substrate we had already used in our previous studies.^53,61^ In a screening performed beforehand, it provided us with the best intensities combined with excellent long-term stability.^61^ A Pt wire as the counter electrode (CE). Both the SERS-active etched Ag wire and the Pt counter electrode were inserted in tailored bushings with a distance of 700 μm. A microscopic picture of the glass body with indicated dimensions is shown in Figure 2C. We designed the detection chamber within the glass body as a square-shaped hollow cavity, facilitating optical focus on the incorporated, flattened SERS substrate. Hence, we constructed an integrated two-electrode electrochemical microflow cell, where the SERS substrate serves as the working electrode (WE), and the Pt wire functions as the counter electrode (CE). The flow direction is designed to encounter the SERS target first, preventing oxidized analytes from interfering with the detection process.

**Figure 2:**
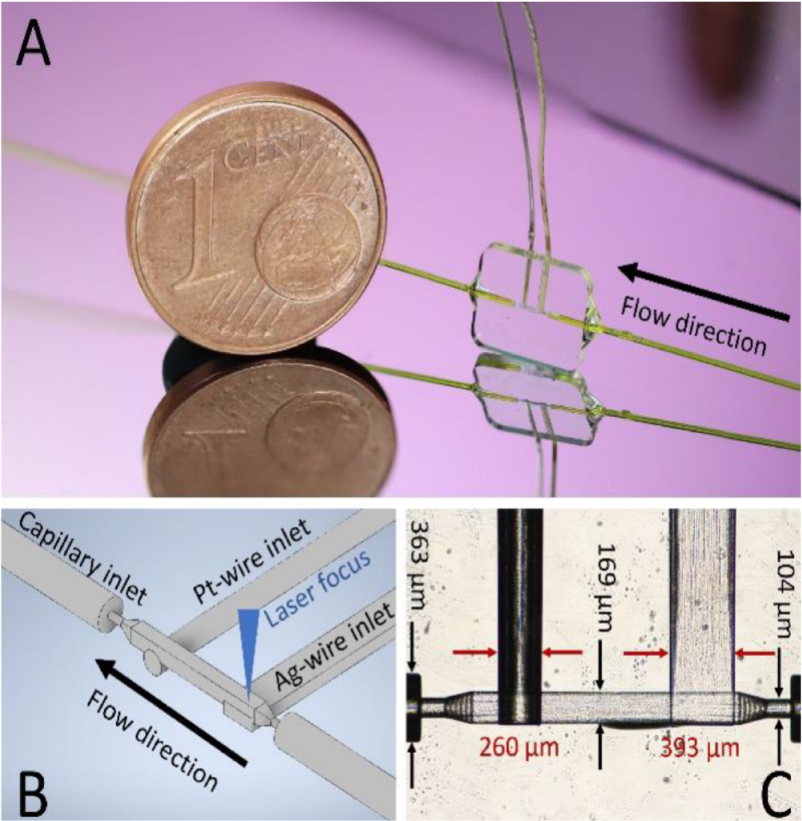
(A) Flow sensor next to a coin for size comparison. (B) Schematic sketch of the glass body indicating inlets for the materials and the future focus spot. (C) Microscopic photograph of the glass body indicating the dimensions.

### Electrochemical Activation of the SERS Substrate

After assembling the developed measurement setup, initial performance testing was executed. First, we studied electrochemical methods to activate the surface of our SERS substrate. The Pt electrode allowed us to apply a reductive potential to the Ag-SERS substrate. In the initial set of experiments, we flushed the device with a solution containing 10 μM crystal violet and 50 mM NaAc/HAc buffer (pH 4.8) in a solvent mixture of 35% MeOH and 65% H_2_O (v/v) at a flow rate of 30 μL/min using a syringe pump. Raman measurements commenced as soon as the analyte solution began filling the chip. We typically used the crystal violet solution with NaAc/HAc buffer for initial activation. The MeOH content reduces surface tension, helping to prevent air bubbles from adhering to the SERS substrate. Crystal violet, as a potent SERS model compound, provides a detectable signal even without prior activation, allowing us to set the focus of the Raman laser to maximize intensity before initiating the cleaning process. As potential solutions for the activation, it is possible to use a broad range of mixtures containing a model substance and some supporting electrolyte. Here, we used conditions close to our later detection under HPLC conditions.

Signal progression over time was monitored at a characteristic analyte band of 1615 cm^– 1^, using an integration time of 1 s and a laser power of 2.5 mW. The resulting signal intensity progression is shown in Figure 3. After starting the recording with the Raman system, we waited until all air bubbles were removed and both the flow and signal intensities had stabilized. In Activation 1, the signal intensity remained at a consistently low level of ∼2.6 au, which is barely sufficient for analysis. After flows and signal intensities stabilized, a looped potential of 0 V for 30 s and −8 V for another 30 s was added.

**Figure 3:**
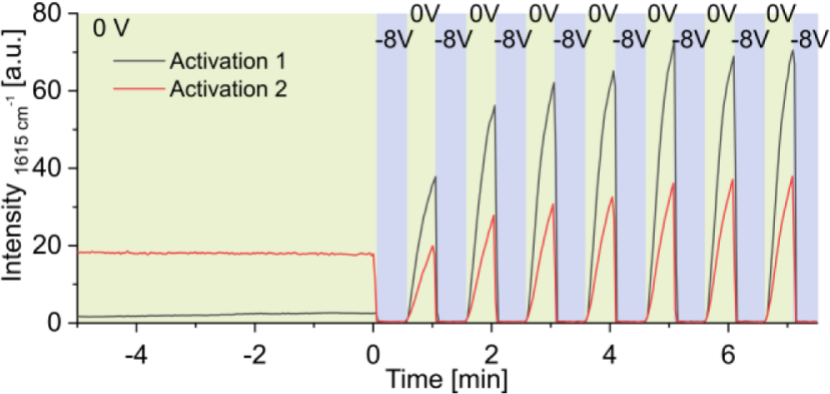
Initial SERS substrate activation of two different sessions. Signal progression of crystal violet over time. 10 μM crystal violet dissolved in 35/75 MeOH/H_2_O at 1615 cm^–1^.

When −8 V was applied, the signal intensity of crystal violet dropped to noise levels due to the reduction of the crystal violet cation (CV^+^) to the corresponding radical (CV^•^), which does not produce a detectable signal. However, when the potential returned to 0 V, we observed a significant amplification of the initial signal. We hypothesize that this amplification is due to the reductive removal of thin oxide or sulfide layers on the Ag nanostructure. We call this process activation. Maximum intensity was observed after five activation cycles, reaching 73.0 au- a 28-fold enhancement compared to the signal intensity without activation. In another run, represented in Figure 3 as Activation 2, we observed an increase in signal intensity from 17.8 au to 37.9 au after seven activation cycles, achieving a 2.1-fold signal enhancement. This activation step is performed daily before starting measurements and remains stable throughout the measurement session. The exact enhancement achieved varies from day to day and depends on the state of the SERS substrate. A substrate that is already in an acceptable condition prior to activation will exhibit a smaller additional growth in signal intensity. Additionally, suppose the SERS substrate performs poorly due to manufacturing variations, aging, or suboptimal laser focus. In that case, the maximum achievable intensity may be lower, resulting in a reduced signal gain via activation. Hence, applying a reductive potential is a fast and effective way to clean and (re)­activate the SERS substrate before measurements. This feature enables our sensor to achieve outstanding longevity. The EC-SERS/HPLC chips maintained their functionality over several months without significant performance drops despite day-to-day intensity variations depending on the substrate’s focus point. However, the exact kinetics of aging require detailed investigation. The limiting factors for the chips’ lifespan included aggressive solvents that dissolve the chip’s glue or corrode the silver electrode, as well as harsh chemicals such as high concentrations of bromide, iodide, or thiosulfate, and oxidative potentials. Chemical influences that provide stable detection in the long term were thoroughly investigated in a prior study.^61^ A brief introduction to our current experiences with various detection conditions, including additional analytes, is provided in SI Chapter 7.

### Model Compound Detection

After these successful initial flow studies, we used the device as a detection cell for true HPLC separations. As a first step, we used malachite green, crystal violet, and rhodamine B as model compounds for HPLC-SERS. Initially, we optimized the separation of these compounds externally using an HPLC setup equipped with a DAD detector and a C18-modified column. To ensure compatibility with electrochemical methods,^61^ we included 50 mM NaAc/HAc in equimolar ratios as a supporting electrolyte. Furthermore, a stable pH value is necessary to achieve a reliable separation of the compound mixture. In a previous study, we noticed that NaAc is a viable supporting electrolyte for EC-SERS detection of crystal violet.^61^ Although Bu_4_NOAc showed to deliver higher intensities, we wanted to stay with supporting electrolytes that also work as common buffers in HPLC. We used an eluent flow rate of 600 μL/min and a solvent ratio of 60/40 MeOH/H_2_O. The compounds with a concentration of 20 μM were injected using a 100 μL sample loop. We observed that a high MeOH content in the eluent reduced the SERS signal intensity of the model compounds to almost undetectable levels. To address this, we introduced a second pump to deliver a makeup flow of H_2_O containing 50 mM NaAc/HAc at a flow rate of 600 μL/min. This adjustment resulted in a solvent mixture at the detection point with a 30/70 MeOH/H_2_O ratio. Our previous study, which thoroughly investigated the influence of the analyte mixture’s chemical composition, enabled us to determine the optimal parameters.^61^ A UV detector was used to cross-check the performance of our EC-SERS detection system. We conducted two comparative series of measurements: (i) Without applied potential: This relied solely on the initial electrochemical surface activation; and (ii) with applied potential. A looped potential program was used, applying 0 V for 8 s, followed by a potential pulse transitioning from 0 V to −8 V over 2 s. In comparison to our previous work,^61^ it was necessary to increase the applied potential from −3.5 V to −8 V to compensate for changes in the overall system size and flow rates. Since no additional signal enhancement is expected from applying potentials due to the buffer/supporting electrolyte,^61^ we applied the periodic potential solely to mitigate the memory effect, resulting in only short pulses with long waiting times of 8s. The results of these measurements are shown in Figure 4. Figure 4A1 displays the Raman chromatogram during the HPLC measurement without applied potentials. The full spectrum is shown as a heatmap. The absorbance at 254 nm (from the UV/vis detector) is plotted alongside selected Raman bands at shifts of 1648 cm^– 1^ and 1615 cm^– 1^ in the chromatogram Figure 4A2. The band at 1648 cm^– 1^ corresponds to the most intense band of rhodamine B, while the 1615 cm^– 1^ band represents the most intense band of malachite green, which can also be used for crystal violet. All compounds are visible in the SERS chromatogram and are baseline-separated. In the heatmap, the full spectra of each compound can be extracted. The model compounds used are known for their strong SERS signal intensity, which is attributed to their high Raman cross sections and strong adsorption to the SERS substrate. However, this strong adsorption hinders desorption after detection, leading to memory effects that are evident as prolonged signal tailing in the chromatogram, as highlighted in Figure 4C1. This tailing is not evident in the upstream recorded UV absorbance trace. The effect is particularly pronounced for rhodamine B. We repeated the measurement with the applied potential program, which looped 8 s of 0.0 V and 2 s transitioning from 0.0 V to −8.0 V. All other parameters were kept the same as in the previous measurement. The results are shown in Figure 4B. The heatmap (Figure 4B1) is displayed alongside a chromatogram (Figure 4B2) containing UV detector data and Raman bands (similar to Figure 4A).

**Figure 4:**
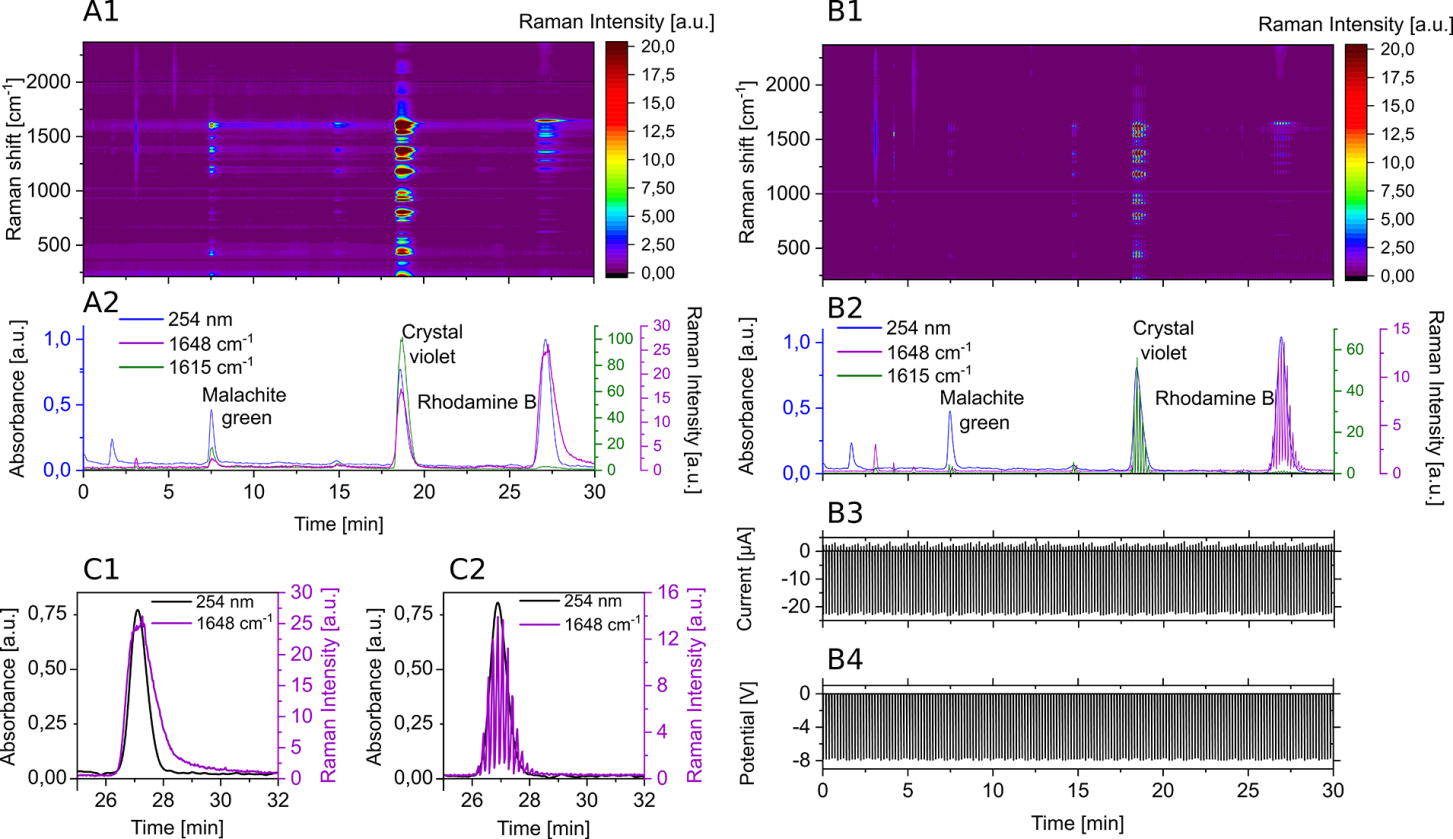
(A) Separation of malachite green, crystal violet, and rhodamine B without applied potentials. (A1) Full Raman spectrum progression over time. (A2) Progression of the UV absorbance and selected Raman band intensities as indicated over time. (B) Separation of malachite green, crystal violet, and rhodamine B with applied potentials (8 s 0.0 V; 2 s from 0.0 V to −8.0 V). (B1) Full Raman spectrum progression over time. (B2) Progression of the UV absorbance and selected Raman band intensities as indicated over time. (B3) Measured current over time. (B4) Measured potential over time. (C) Comparison of Raman signal tailing of rhodamine B between measurements without (C1) and with applied potential program (C2). Raman settings for all measurements: 1 s integration time; 2.5 mW laser power.

The measured current (Figure 4B3) and potentials (Figure 4B4) are also plotted to correspond to the chromatogram. The signal intensities of the Raman signal are interrupted each time the potentials goes to −8 V likely due to the reduction of the model compounds to species with lower Raman signal intensities. Overall, the Raman intensities appear reduced compared to the measurement without applied potentials. Figure 4C shows a zoomed-in chromatogram of rhodamine B elution, comparing retention profiles obtained with applied potentials (C2) versus without applied potentials (C1). The UV absorbance and the most intense Raman band of rhodamine B are plotted. Without applied potential, the Raman signal exhibits clear tailing which can be attributed to the memory effect. With applied potential, the Raman signal returns to baseline alongside the UV absorbance. Therefore, applying potentials can suppress the memory effect, which is valuable in scenarios where analytes elute closely. However, this suppression is situational and not universal for all substances. Additional data from these experiments are provided in SI Chapter 2, which includes zoomed-in views of the eluting analytes and the extracted spectra. Furthermore, SI Chapter 3 includes another separation of crystal violet, malachite green, and rhodamine 6G, which provides deeper insight into reproducibility.

### Reproducibility of HPLC EC-SERS

To further evaluate the reproducibility of our newly developed technique, a series of experiments was performed using a 10 μM crystal violet solution in the EC-SERS HPLC system. Signal reproducibility was assessed by performing three replicate injections under two conditions: without applied potentials (Figure 5A) and with applied potentials (Figure 5B). All experiments within each set were conducted consecutively without intermediate substrate activation. To compare the signal intensities, both the peak intensity (maximum peak height) and peak area were analyzed. Mean values and relative standard deviations were calculated, and the corresponding data are presented in Table 1. It is clearly evident that measurements performed without an applied potential program exhibit significantly higher signal intensities compared to those with applied potentials. However, in the absence of applied potentials, a drift toward lower intensities is observed across repeated measurements, accompanied by higher relative standard deviations. In contrast, when potentials are applied, we propose that consistent reductive cleaning of the SERS substrate suppresses fluctuations in signal intensity, resulting in lower standard deviations and no significant drift at the cost of lower overall intensities.

**Figure 5:**
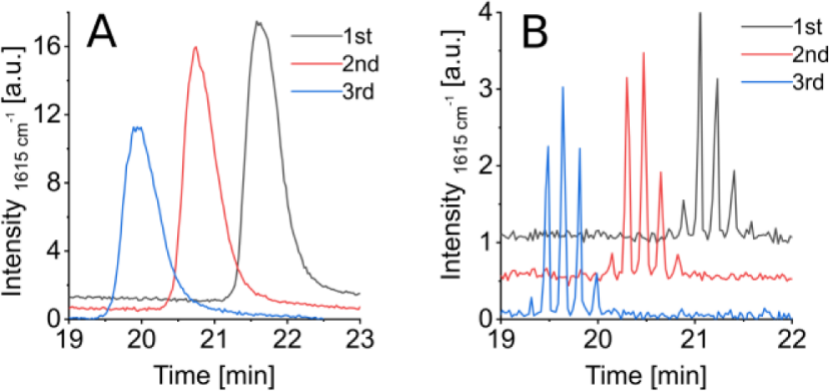
Repeated measurements of crystal violet under HPLC conditions in consecutive runs. (A) Without potential program. (B) With underlying potential program (8 s 0.0 V; 2 s from 0.0 V to −8.0 V). Sample: 100 μL of 10 μM crystal violet dissolved in 35/75 MeOH/H_2_O at 1615 cm^–1^. Raman settings: 1s integration time; laser power: 5 mW.

**Table 1:** Reproducibility of EC-SERS Detection of Crystal Violet in an HPLC Setting without Applied Potentials (A) and with Applied Potentials (B)

	No potential (A)		Potential applied (B)	
	Peak height [a.u.]	Peak area [a.u. · s]	Peak height [a.u.]	Peak area [a.u. · s]
first	16.5	382	3.31	16.1
second	15.5	345	2.97	18.8
third	11.3	275	3.02	18.6
mean	14.4	334	3.10	17.8
RSD [%]	15.6	13.2	4.85	6.82

### EC-HPLC SERS for the Detection of B-Vitamins

After the proof-of-concept study with model compounds, we applied the method to more real-world samples, exemplified by the analysis of a mixture of Vitamin B9 (folic acid) and B12 (cyanocobalamin). We used an HPLC flow rate of 400 μL/min of eluent consisting of 30/70 MeOH/H_2_O containing 0.1% HAc to separate these substances. 100 μL/min MeOH containing 0.25 M Bu_4_NOAc as supporting electrolyte was used as makeup flow. It was necessary to switch to Bu_4_NOAc as the supporting electrolyte since NaAc/HAc based buffers did not provide sufficient intensities. We did not observe significant differences in SERS signal intensities during method optimization when varying the solvent composition regarding the MeOH/H_2_O ratio. We used a 20 μL sample loop to inject the solution, which matched the eluent, containing 1 mM cyanocobalamin and 6.8 μM folic acid. To detect the substances, we adjusted the laser and readout settings to higher sensitivities. We ended up with a laser power of 6.3 mW and an integration time of 3 × 1 s, allowing us to record one frame every 3.3 s. An electrical potential program was applied, looping at 0 V for 2 s and then sweeping from 0 to −8 V over 8 s. Figure 6 shows the resulting chromatogram alongside the voltage program. The heatmap in Figure 6A1 displays the progression of the Raman spectra over time. We extracted the signal intensity at a wavenumber of 1593 cm^– 1^, representing both analytes and plotted its progression alongside the UV detector output at 360 nm in the chromatogram in Figure 6A2. Figure 6A3 represents the measured current over time, and Figure 6A4 represents the applied potential. It is visible that the recorded Raman signal intensities fluctuate with the applied potential. As previously discussed, the measurement was performed immediately after activating and calibrating the system with a crystal violet solution to achieve the best intensities from the analytes. In the first 3 min of the measurement, the slowly decreasing signal from the crystal violet memory effect is visible. Cyanocobalamin eluted after 5.3 min, producing significant absorbance and a Raman spectrum. At 7.5 min, folic acid began to elute, resulting in a significant Raman spectrum but no absorbance due to its low concentration. The extracted Raman spectra of both compounds are displayed in Figures 6-C1 and 6-C2. Zoomed-in graphs of the eluting analytes, along with an accompanying discussion, are provided in Figure S6. The zoomed-in graphs make it possible to correlate the recorded spectra with the applied potential. SI Chapter 4 contains a reproduction of the measurement. Figure 6B1 shows the Raman signal progression for the separation of cyanocobalamin and folic acid under identical conditions but without applied potentials. The associated chromatogram in Figure 6B2 includes the UV absorbance at 360 nm and the extracted Raman intensities at 1593 cm^– 1^. A weak signal at 7.5 min, related to folic acid elution, is detectable. However, extracting meaningful Raman spectra for these substances was challenging due to the low signal intensities. After the measurement, we confirmed that all calibrations were performed correctly to verify these results. We conclude that including electrochemical methods in our HPLC-SERS system enables the detection of compounds that would otherwise remain virtually invisible under the chosen experimental conditions. We hypothesize that the electrical potential promotes the adsorption of the substances onto the SERS substrate, which suffers from low contact time between analyte and substrate in an HPLC context.

**Figure 6:**
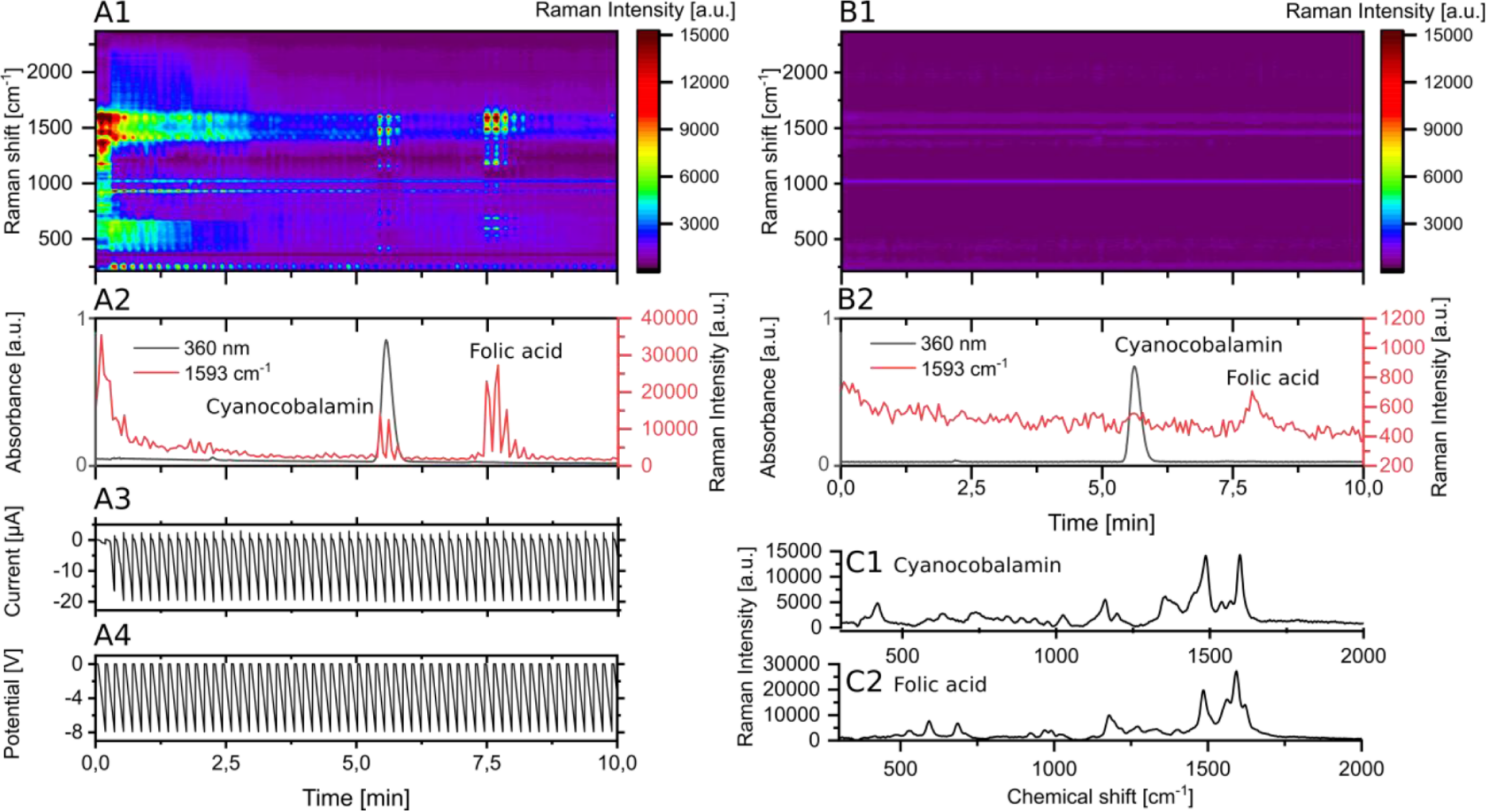
(A) Separation of cyanocobalamin and folic acid with applied potentials (2 s 0.0 V; 8 s from 0.0 V to −8.0 V). (A1) Full Raman spectrum progression over time. (A2) Progression of the UV absorbance and selected Raman band intensities as indicated over time. (A3) Measured current over time. (A4) Measured potential over time. (B) Separation of cyanocobalamin and folic acid without applied potentials. (B1) Full Raman spectrum progression over time without applied potentials. (B2) Progression of the UV absorbance and selected Raman band intensities as indicated over time without applied potentials. (C) Extracted Raman spectra of cyanocobalamin (C1) extracted from (A1) and folic acid (C2) extracted from (A1). Raman settings for all measurements: 3 × 1 s integration time; 6.3 mW laser power.

## Conclusion

We developed an HPLC-compatible microflow cell that integrates an Ag-SERS substrate with a Pt counter electrode, allowing for the use of EC-SERS as a detection method in HPLC. The application of potentials enabled a substantial increase in the signal intensities of certain compounds that would have otherwise remained virtually undetectable and allowing for the suppression of memory effects. This signal enhancement allows us to expand the scope of HPLC-SERS beyond model compounds to real-life substances. Furthermore, it substantially extends the lifetime of our SERS substrates. We believe that EC-SERS detection in HPLC represents an innovative step toward making the promising technique of SERS more accessible for routine analytical applications. Our future work will focus on lowering the limits of detection, quantification, and reproducibility to allow for consistent method development.

## Supplementary Material



## Notes

Detailed description of experimental setup; details of model compound detection; separation of crystal violet, malachite green, and rhodamine 6G; details of B-Vitamin detection; details about data processing (PDF)
