# Supplementary material for: A Microfluidic Device to Realize Electrochemically Controlled SERS Detection in HPLC
Source: Anal Chem. 2025 Jun 23;97(25):13628–36. doi: 10.1021/acs.analchem.5c02232 (PMC12224159; doi:10.1021/acs.analchem.5c02232)
Supplement: Supplementary file 1 [file ac5c02232_si_001.pdf]

Supplementary Information

A Microfluidic Device to Realize Electrochemically Controlled SERS Detection in HPLC

Maximilian E. Blaha[a], Julius Schwieger[a], Rico Warias[a], Anish Das[a], Matthias Polack[a], and Detlev Belder[a]

[a] Institute for Analytical Chemistry, Leipzig University, Linnéstraße 3, 04103 Leipzig, Germany.

1. A more detailed description of the electronics part

The fluidics and optics are already explained in the main paper. Here, the electronics are described. A schematic sketch of the experimental setup involving the electronics is shown in Figure S1. The roles of each involved input and output of the I/O device are provided in the description of the figure.

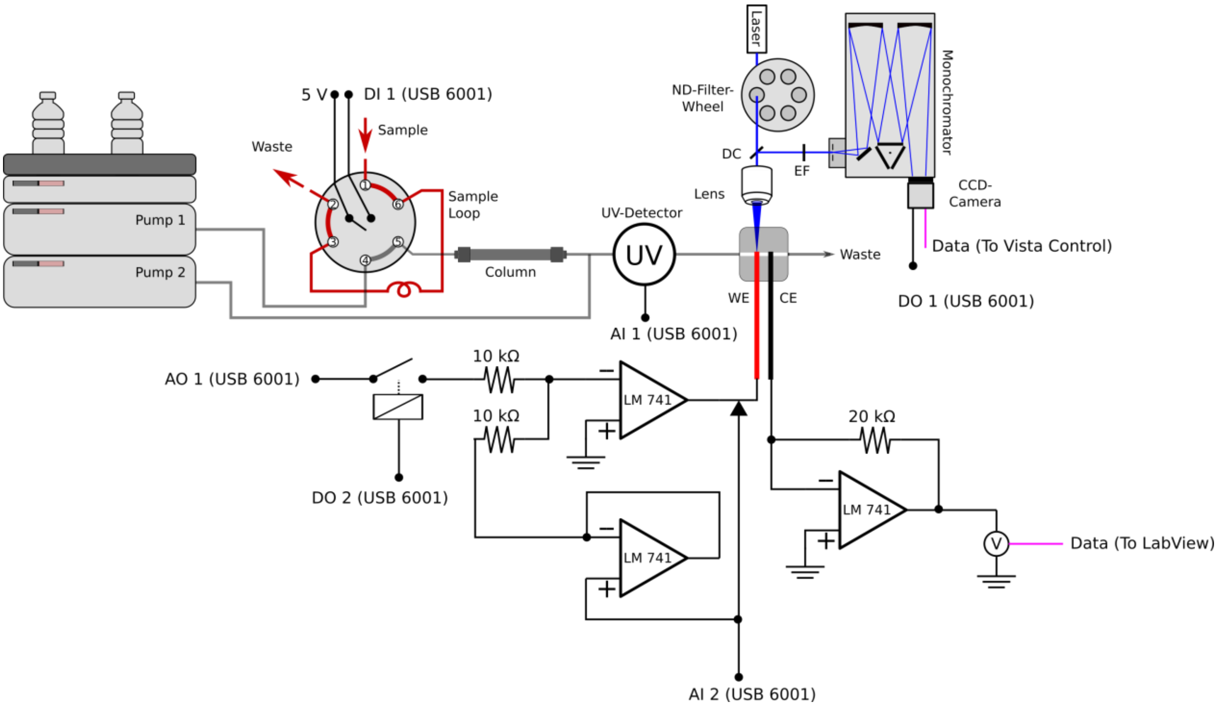

Figure S1: Schematic sketch of the experimental setup including the electronics used to drive the chip. AO 1: Analog Output, programmed to provide the applied voltage program to the circuit. DO 1: Triggers the CCD camera to start the measurement. DO 2: Triggers the relay, enabling the circuit. AI 1: Reads out the monitor of the UV detector (Spectra 100, Spectra Physics, CA, USA). AI 2: Reads out the potential applied to the working electrode. DI 1: Digital Input, which reads out the position of the hexaport valve. When injection is performed with the hexaport, 5 V are applied to the input, and the measurement is started. Data 1: Data output from the CCD camera (ProEM, Princeton Instruments, USA) is read out via the software VistaControl V4.8.3 (S&I Spectroscopy & Imaging GmbH). Data 2: Data output from the digital precision multimeter (DMM6500, Keithley, Ohio, USA), which is used to read out the current monitor. Current is calculated using Ohm's law.

We used a self-made potentiostat in a two-electrode setup, as it provided stable performance over long-duration measurements under varying conditions. We developed a program based on LabVIEW 2017 (National Instruments, USA), which allowed us to apply a defined repetitive voltage program via an I/O device (USB-6001, National Instruments, USA). The signal was amplified using an LM741 (Texas Instruments, TX, USA) operational amplifier in its voltage follower configuration, powered by a standard laboratory power supply (DC Dual Power Supply 6145, PeakTech, Ahrensburg, Germany). The output of the op-amp was connected to the counter electrode (Pt-wire) of the chip. The working electrode was connected to another LM741 op-amp functioning as a current monitor. For the current monitoring circuit, we included

a 20 kΩ resistor to calculate the current using Ohm's law and measured the output voltage with a precision multimeter (DMM6500, Keithley, Ohio, USA). The LabVIEW program read out the applied voltage (via the I/O device), the current monitor voltage (via the multimeter), and the UV detector (via the I/O device). It also allowed us to set triggers for the Raman system and read the trigger from the hexaport valve to synchronize the Raman system with the electrochemical setup and the data acquisition.

2. Additional Details of model compound detection with applied potentials during HPLC.

Figure S2 provides additional depictions of data extracted from the model compound detection with applied potentials, as described in the main paper. The figure includes zoomed-in views of the eluting analytes malachite green (Figure S2-A), crystal violet (Figure S2-B), and rhodamine B (Figure S2-C). The data features the intensity progression as a heatmap (Figures S2-A1, S2-B1, and S2-C1), the intensity progression of the Raman bands at 1648 cm^{-1} and 1615 cm^{-1} alongside the UV-detector readout (Figures S2-A2, S2-B2, and S2-C2), the corresponding current progression (Figures S2-A3, S2-B3, and S2-C3), and the applied potential progression (Figures S2-A4, S2-B4, and S2-C4).

The black lines indicate the points of highest intensity during the voltage sweeps. It is evident that the intensities decrease with each pulse and that the highest intensities occur just before the -8 V pulses.

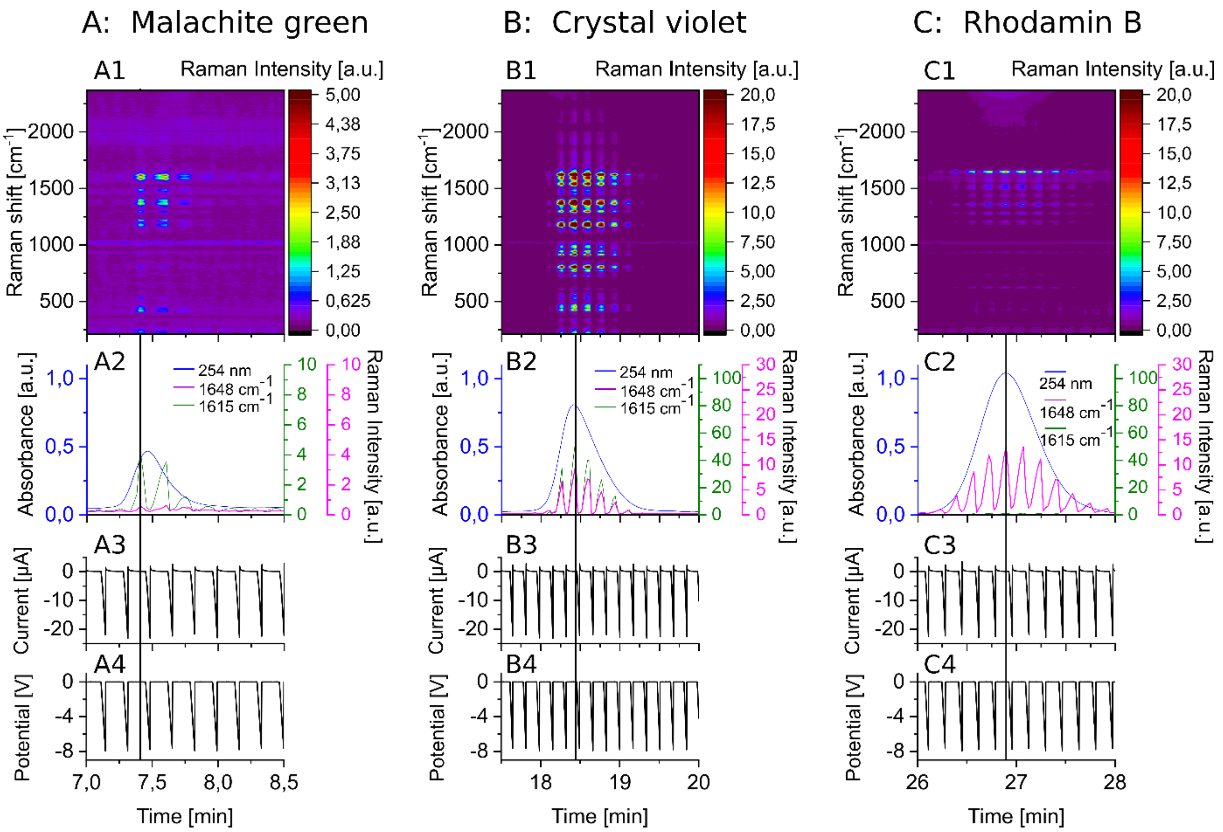

Figure S2: Isolated signal progressions of the Raman signals during HPLC measurement together with the UV detector readout and the applied potentials and electrical current for malachite green (A), crystal violet (B), and rhodamine B (C). Mobile Phase: 50 mM NaAc/HAc; 60/40 MeOH/H_2O; 600 µL/min Makeup-flow: 50 mM NaAc/HAc; H_2O; 600 µL/min; Laser: 473 nm; 2.5 mW; 5 x 0.2s Integration time. Analytes: 20 µM; 100 µL. Column: C18. Potential: 0V 8s; 0 to -8V 2s.

Figure S3-A shows the extracted Raman spectra from the measurement with applied potentials, taken at the points of highest intensity for malachite green (Figure S3-A1, 7.41 min), crystal violet (Figure S3-A2, 18.44 min), and rhodamine B (Figure S3-A3, 26.90 min). Figure S3-B displays the extracted Raman spectra from the model compounds at the points of highest intensity from the measurement without applied potentials, as described in the main paper. Shown are the spectra for malachite green (Figure S3-B1, 7.56 min), crystal violet (Figure S3-B2, 18.61 min), and rhodamine B (Figure S3-B3, 27.27 min).

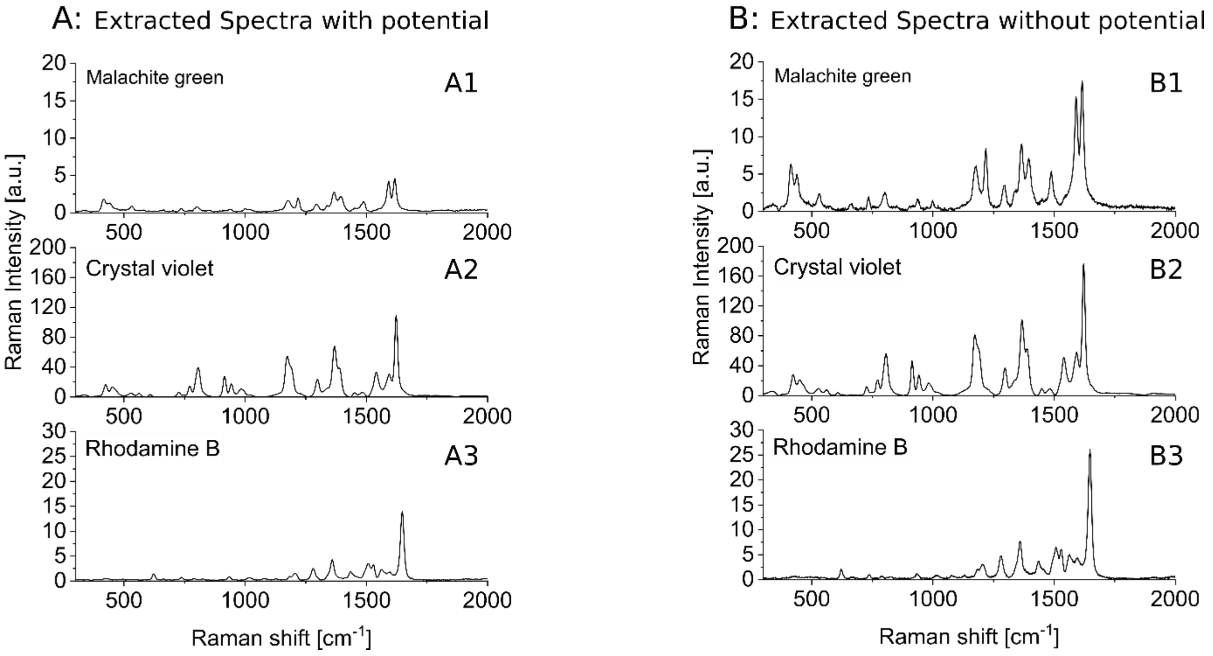

Figure S3: Extracted Raman spectra comparative from the measurement with potential program (A) and the measurement without applied potentials (B). Mobile Phase: 50 mM NaAc/HAc; 60/40 MeOH/H_2O; 600 µL/min Makeup-flow: 50 mM NaAc/HAc; H_2O; 600 µL/min; Laser: 473 nm; 2.5 mW; 5 x 0.2s Integration time. Analytes: 20 µM; 100 µL. Column: C18. Potential: 0V 8s; 0 to -8V 2s. Most intense signal intensities of the chromatogram were used.

3. Separation of crystal violet, malachite green and rhodamine 6G

To underscore the robustness of our newly developed EC-SERS HPLC detection technique, we present additional HPLC measurements demonstrating the separation of crystal violet, malachite green, and rhodamine 6G. In the presented measurements, we show the separation of crystal violet, malachite green, and rhodamine 6G. As concentrations, we used 10 µM solution of the analytes dissolved in water. As a mobile phase, we used a solution of 60/40 MeOH/H_2O containing 50 mM NaAc/HAc buffer at a flow rate of 600 µL/min. As a make-up flow, we used 50 mM NaAc/HAc dissolved in water at a flow rate of 300 µL/min. This results in a total flow rate of 900 µL/min, delivering a final composition of 40/60 MeOH/H_2O at the SERS substrate. The applied potential program consisted of an 8-second hold at 0 V followed by a 2-second linear sweep from 0 V to -8 V. The measurement was carried out twice. The chromatograms containing the progression of the full Raman spectrum of both measurements are displayed in Figure S4-C and Figure S4-D. For rhodamine 6G, fluorescence is visible at wavenumbers above 1600 cm^{-1}. In the chromatogram shown in Figure S4-A, the progression of the Raman intensity at 1615 cm^{-1} and in Figure S4-B the progression of the Raman intensity at 1645 cm^{-1} is shown for both runs. Based on these chromatograms, we extracted the peak height and the peak area as markers for the intensity. For malachite green and crystal violet, we used the 1615 cm^{-1} indicative band, and for rhodamine 6G, we used 1645 cm^{-1} as the indicative band for determining peak height and peak area. The extracted intensities, their mean values, and their percentage differences are shown in Table S1. Differences of 24% to 33% for the peak height and 12% to 42% for the peak area indicate that further optimizations are required for reliable quantification.

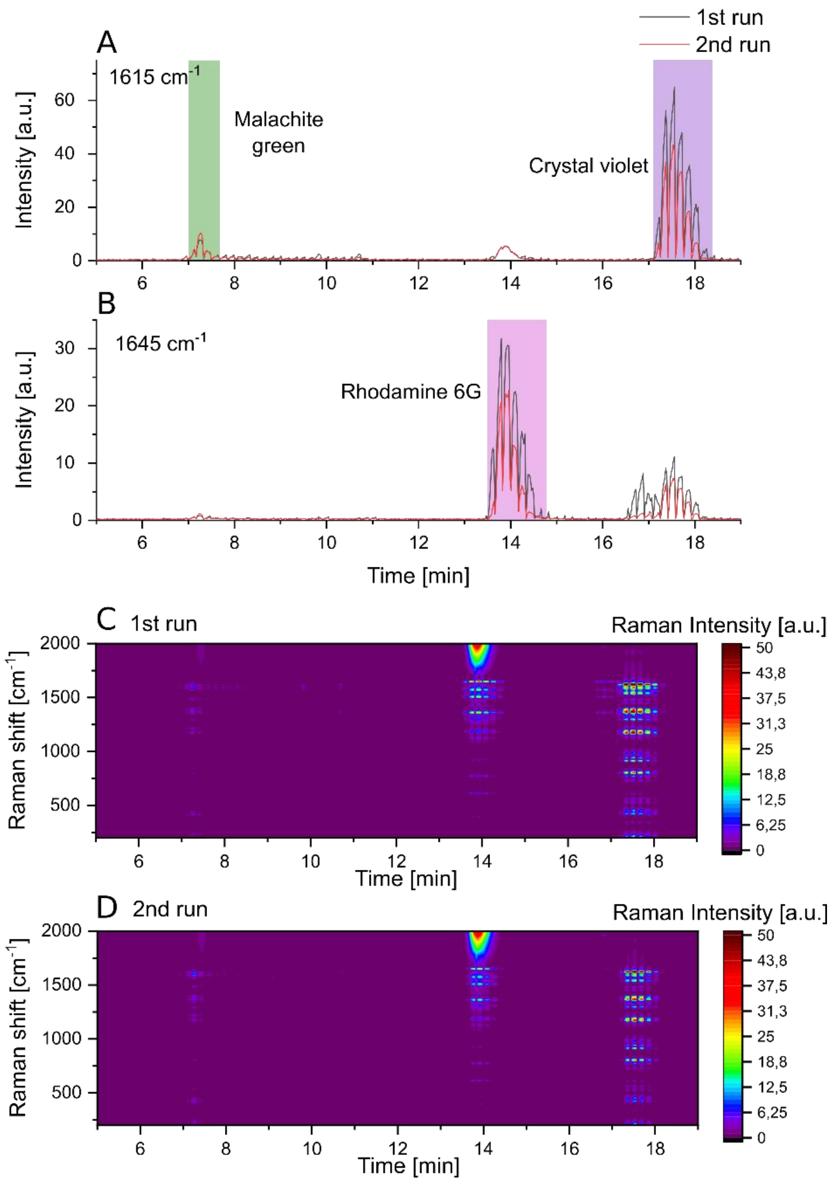

Figure S4: Two consecutive separations of malachite green, crystal violet, and rhodamine 6G with applied potentials. A: chromatogram at 1615 cm-1. B: chromatogram at 1645 cm-1. C: Chromatogram of the full Raman spectrum of the first run. D: Chromatogram of the full Raman spectrum of the second run. Settings for all measurements: Applied potentials (8 s 0.0 V; 2 s from 0.0 V to -8.0 V). Raman Settings: 5 x 0.2 s integration time. 2.5 mW Laser power. Samples: 10 µM dissolved in H_2O. Mobile Phase: 50 mM NaAc/HAc; 60/40 MeOH/H_2O; 600 µL/min Makeup-flow: 50 mM NaAc/HAc; H_2O; 300 µL/min.

Table S1: Two consecutive separations of malachite green, crystal violet, and rhodamine 6G were performed with applied potentials. Settings are as in Figure S4. Peak height and peak area were extracted for each run. Mean values and the percentual difference between the measurements are provided.

Peak height (band)	Run 1 [a.u.]	Run 2 [a.u.]	Δ [%]	mean
MG (1615 cm^{-1})	7,8	10,3	24	9,0
R6G (1645 cm^{-1})	31,9	22,7	29	27
CV (1615 cm^{-1})	65,0	43,4	33	54

Peak Area (band)	Run 1 [a.u.·s]	Run 2 [a.u.·s]	Δ [%]	mean
MG (1615 cm^{-1})	88,8	101	12	95
R6G (1645 cm^{-1})	608	341	44	474
CV (1615 cm^{-1})	1155	668	42	911

4. Additional details of B-Vitamin detection with applied potentials during HPLC

Figure S5 displays a 3D contour plot of the performed HPLC with EC-SERS detection of B-Vitamins. The slowly decreasing memory effect of the calibration substance is visible at the beginning of the measurement. Vitamin B12 (cyanocobalamin) elutes after 5 minutes, while vitamin B9 (folic acid) elutes after 7.5 minutes.

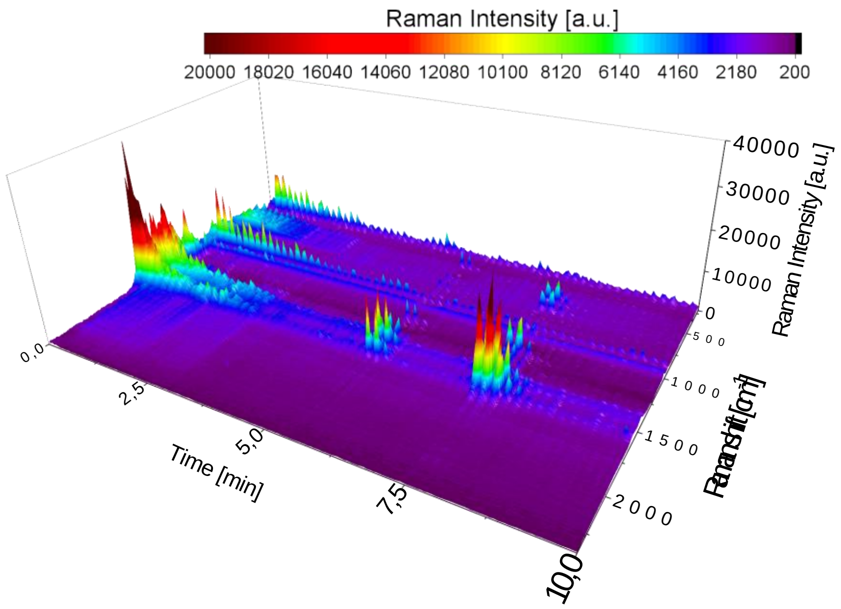

Figure S5: 3D contour plot of the performed HPLC with EC-SERS detection of B-Vitamins. Mobile Phase: 0.1% HAc; 30/70 MeOH/H_2O; 400 µL/min. Makeup-flow: 250 mM Bu_4NOAc; MeOH 100 µL/min; Laser: 473 nm; 6.3 mW; 3 x 1s Integration time. Analytes: 1 mM B12; 6.8 µM B9; 20 µL Column: C18. Potential: 0V 2s; 0 to -8V 8s.

Figure S6 provides additional representations of data extracted from vitamin detection experiments with applied potentials, as described in the main paper. The figure includes zoomed-in views of the eluting analytes cyanocobalamin (Figure S6-A) and folic acid (Figure S6-B). The data features the intensity progression as a heatmap (Figures S6-A1 and S6-B1), the intensity progression of the Raman bands at 1593 cm^{-1} alongside the UV-detector readout (Figures S6-A2 and S6-B2), the corresponding current progression (Figures S6-A3 and S6-B3), and the applied potential progression (Figures S6-A4 and S6-B4).

A black line is included to align the highest intensity frame with the applied potential and current. It can be observed that the highest intensities occur during the sweeps, indicating that the applied potential plays a crucial role in enhancing signal intensities.

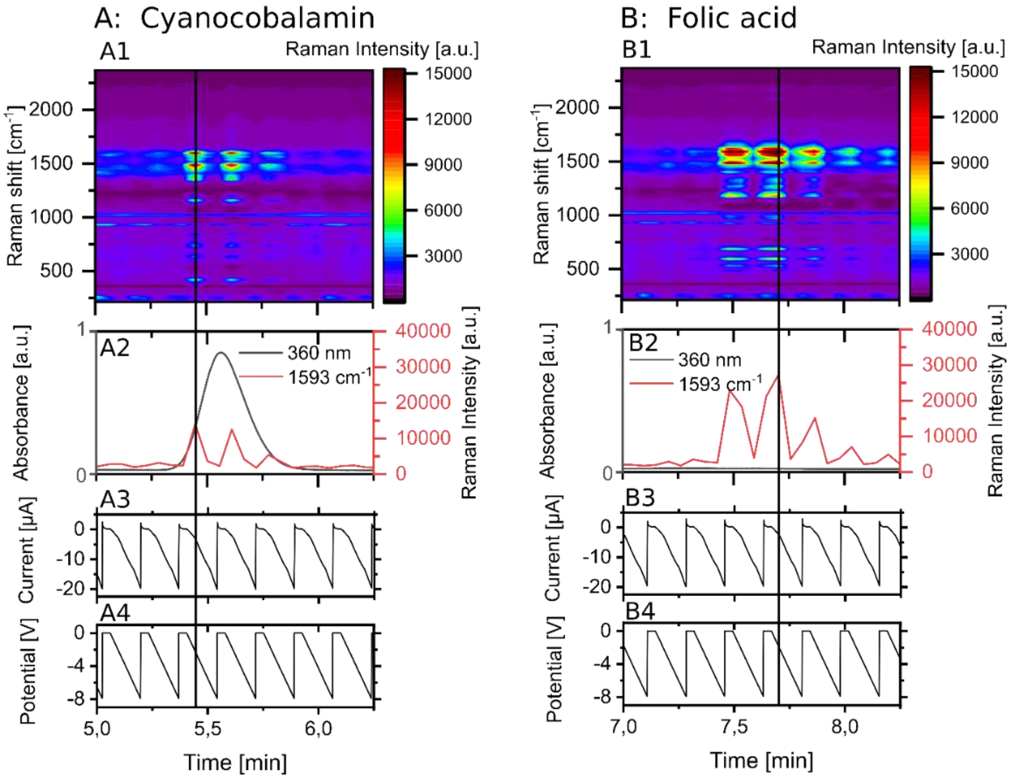

Figure S6:Isolated signal progressions of the Raman signals of B-Vitamins during HPLC measurement together with the UV detector readout and the applied potentials and electrical current for cyanocobalamin (A) and folic acid (B). Mobile Phase: 0.1% HAc; 30/70 MeOH/H_2O; 400 µL/min. Makeup-flow: 250 mM Bu_4NOAc; MeOH 100 µL/min; Laser: 473 nm; 6.3 mW; 3 x 1s Integration time. Analytes: 1 mM B12; 6.8 µM B9; 20 µL Column: C18. Potential: 0V 2s; 0 to -8V 8s.

To ensure the reproducibility of our findings, we repeated the HPLC separation immediately after the initial run without intermediate activation or cleaning of the SERS substrate. The resulting 3D contour plot is shown in Figure S7. The repeated measurement reveals no memory effect from the calibration substance at the start of the run. However, a general decrease in signal intensity was observed for both analytes: Vitamin B12 (cyanocobalamin, eluting at ~5 minutes) and Vitamin B9 (folic acid, eluting at ~7.5 minutes). For easier comparison, Figure S8 displays the intensity profiles of both chromatograms at 1593 cm^{-1}, clearly illustrating the reduction in signal intensity between the two measurements.

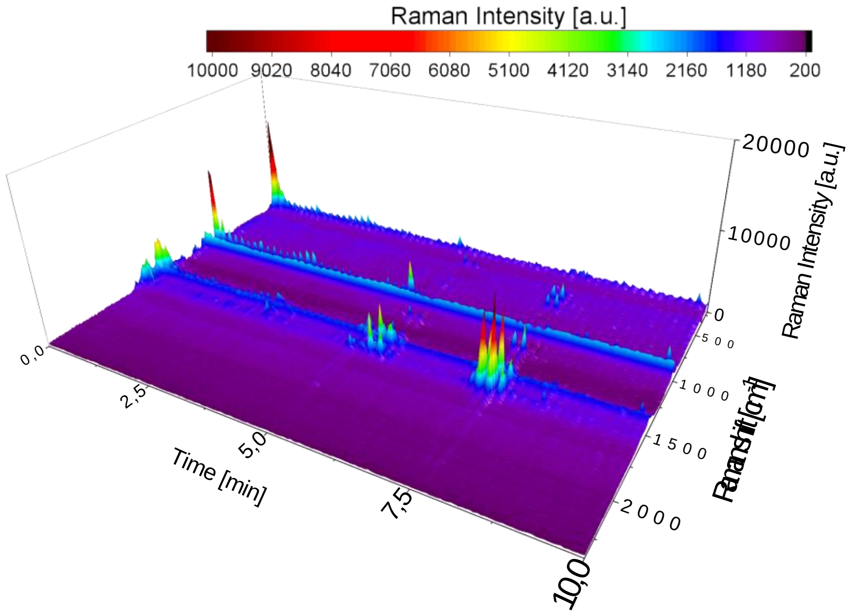

Figure S7: *3D contour plot of the performed HPLC with EC-SERS detection of B-Vitamins. Repeated measurement. Mobile Phase: 0.1% HAc; 30/70 MeOH/H_2O; 400 µL/min. Makeup-flow: 250 mM Bu_4NOAc; MeOH 100 µL/min; Laser: 473 nm; 6.3 mW; 3 x 1s Integration time. Analytes: 1 mM B12; 6.8 µM B9; 20 µL Column: C18. Potential: 0V 2s; 0 to -8V 8s.*

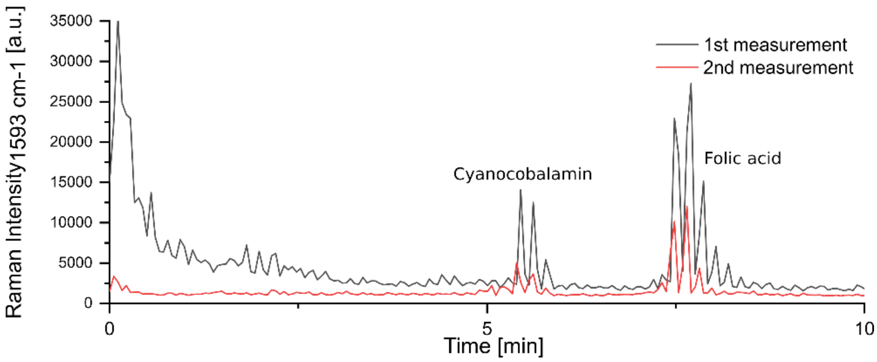

Figure S8: Comparison of chromatograms from the separation of folic acid and cyanocobalamin detected via EC-SERS. Black: Measurement performed immediately after calibration and activation of the SERS substrate. Red: Repeated measurement without intermediate reactivation of the SERS substrate.

5. Data processing

The Raman spectra were processed using the software VistaControl V4.8.3 (S&I Spectroscopy & Imaging GmbH), where smoothing and background correction were performed. Correcting the background is crucial, as the background signal varies with the applied potential. This correction allows for a clear distinction between variations in background intensity and genuine changes in the Raman signal intensity.

6. Model compounds and pharmaceutical compounds measured in one session

To assess whether competitive adsorption of model compounds (crystal violet) might hinder or compromise the detection of non-model compounds (Adenine and folic acid), we performed a measurement simulating a mixture of both. We set up a flow injection measurement to simulate an HPLC experiment.

To do so, we modified the setup by removing the column and injecting each sample separately via the hexaport valve. The resulting data, along with all experimental parameters, are shown in Figure S9, which includes the full Raman signal progression (Figure S9-A), the signal progression of two selected Raman bands (Figure S9-B), the measured current (Figure S9-C), and the applied potential (Figure S9-D).

It is clearly visible that adenine shows comparatively low intensities, despite being used at a relatively high concentration (1 mM). In contrast, folic acid (10 µM) exhibits intensities comparable to those of crystal violet (10 µM). Notably, memory or carryover effects do not compromise the readouts under the conditions applied. In Figure S10, a spectrum for each analyte was extracted from the Raman signal at its point of highest intensity to demonstrate that the substances can be reliably identified.

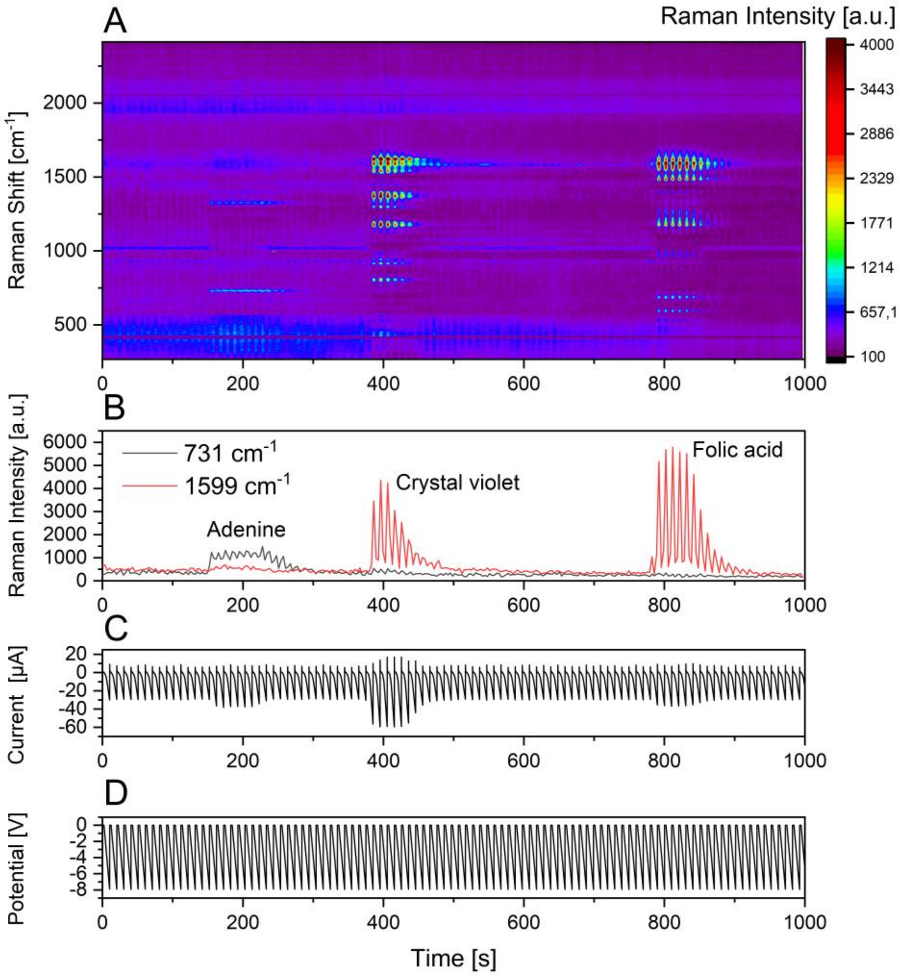

Figure S9: A: Flow injection measurement of Adenine, crystal violet and folic acid with applied potentials (2 s 0.0 V; 8 s from 0.0 V to -8.0 V). A: Full Raman spectrum progression over time. B: Progression of selected Raman band intensities as indicated over time. C: Measured current over time. D: Measured potential over time. Raman Settings: 3 × 1 s integration time. 5 mW Laserpower. 473 nm. Eluent: 20/80 MeOH/H_2O. Flowrate: 200 µL/min. Samples: Sample Volume: 200 µL. Folic acid: 10 µM dissolved in 2/98 MeOH/H_2O. Adenine: 1mM dissolved in H_2O. Crystal violet: 10 µM dissolved in H_2O containing 50 mM Bu_4NOAc. Make up flow: 50 µL/min H_2O containing 250 mM Bu_4NOAc.

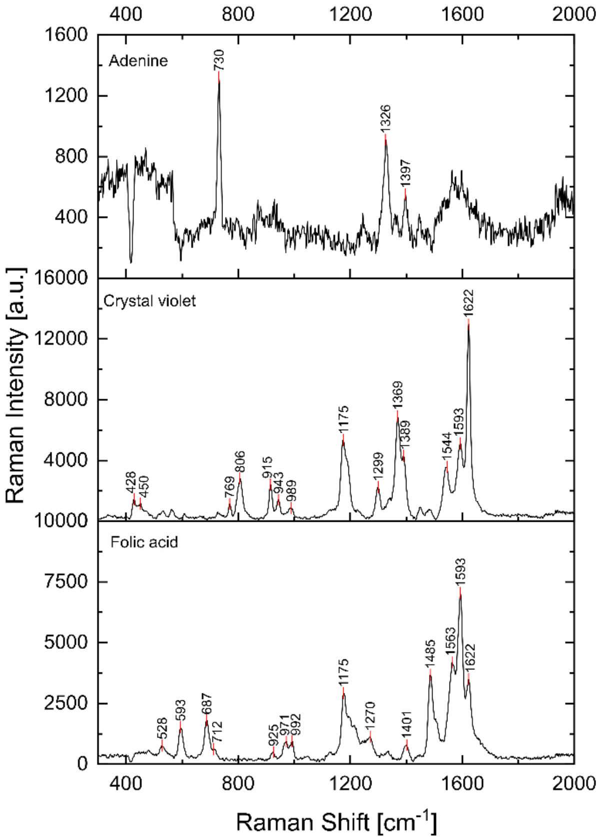

Figure S10: Extracted spectra from the flow injection measurement displayed in Figure S9. Displayed are the frames with the highest peak intensity for each analyte.

7. Chemical influences on the detectability of compounds and long-term stability of the chip.

Every detection technique requires long-term stability and a certain degree of universality to become a viable option for routine analytical applications. Based on our current experimental progress, we share the following insights regarding the chemical and long-term stability of the SERS substrate, as well as the detectability of compounds - extending the findings of our previous study.[1]

To this end, we assessed the compatibility of the substrate with solvents commonly employed in RP-HPLC, including water, methanol, and acetonitrile. In terms of chemical stability, none of these solvents significantly compromised the functional longevity of the SERS substrate. However, we observed that the physical integrity of the chip is affected by acetonitrile. Specifically, acetonitrile induces swelling in the adhesive used during chip fabrication, eventually resulting in chip failure.

Within the current setup, acetonitrile-based solutions can be used only for a limited duration of up to 3 hours. To demonstrate the applicability of the chip under these conditions, we performed a continuous flow measurement using a 10 µM solution of Emtricitabine (BLDpharm, Germany) dissolved in 90/10 MeCN/H_2O containing 50 mM Bu_4NOAc. The solution was delivered through the chip at a flow rate of 30 µL/min using a syringe pump (PHD 2000, Harvard Apparatus, USA). A cyclic potential program was applied, ramping from 0 V to –10 V over 10 seconds, followed by 0 V for 10 seconds, in a repeating loop. The time-

resolved Raman signal progression is presented in Figure S11, with clear evidence of a potential-dependent Raman response.

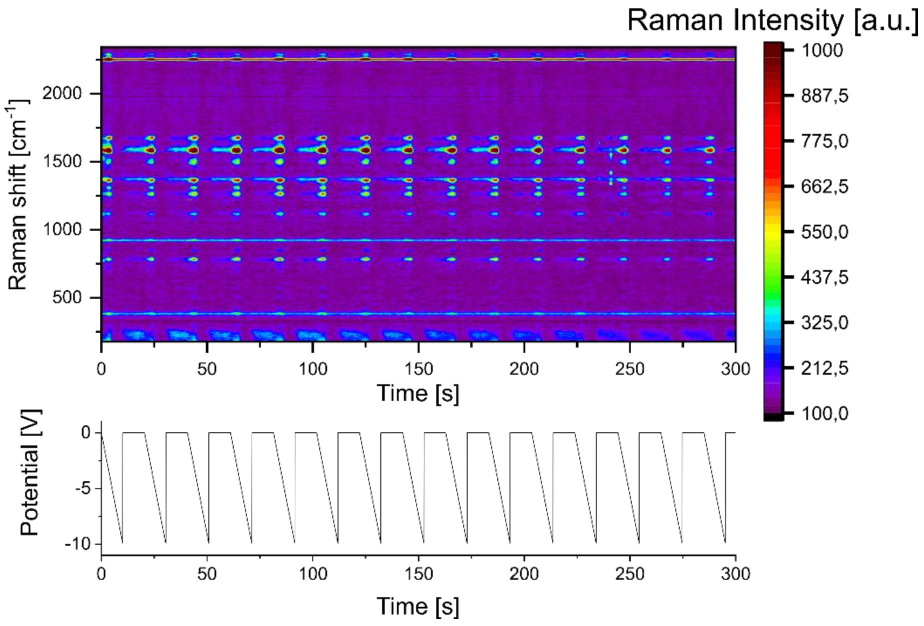

Figure S11: EC-SERS signal progressions of Emtricitabine 10 µM concentration and dissolved in 90/10 MeCN/H_2O containing 50 mM Bu_4NOAc. Spectra were recorded under constant flow of 30 µL/min. Integration time: 1s. Laser power: 2.5 mW. Potential program: 10s from 0 to -10 V followed by 10 s with 0V.

The Raman spectrum extracted at 3.3 seconds is shown in Figure S12. Raman bands corresponding to acetonitrile are indicated in blue, while those associated with Emtricitabine are highlighted in light red. The diagram demonstrates that HPLC measurements are theoretically feasible using acetonitrile-based solutions, underscoring the robustness and stability of our EC-SERS setup under these conditions.

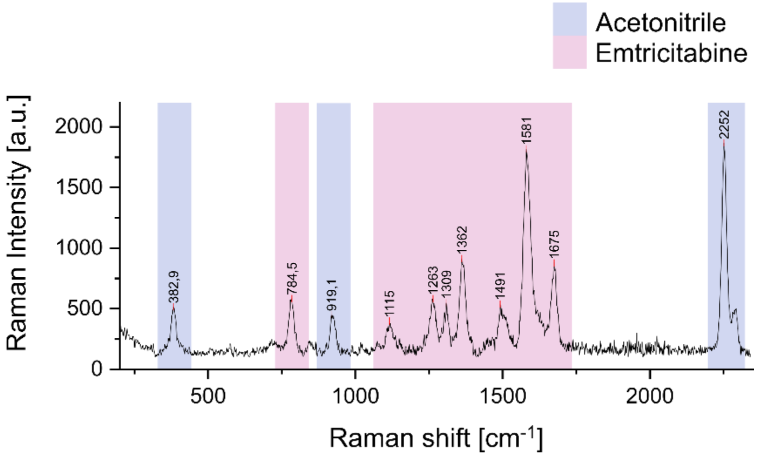

Figure S12: Extracted Raman spectrum from Figure 119 at 3.3 s (corresponding to 3.3 V). Bands originating from the acetonitrile background are indicated in blue, while bands corresponding to emtricitabine are marked in red.

The detectability of analytes varies significantly depending on the solvent used, as solvents can influence the adsorption of analytes onto the SERS substrate and thereby affect signal intensity. From an electrochemical perspective, acetonitrile offers a broader electrochemical window, allowing the induction of spectroelectrochemical changes in analytes that are not feasible with solvents that degrade at lower potentials, such as water or methanol. However, acetonitrile also introduces a strong background signal (most prominent at 380 cm^{-1}, 918 cm^{-1}, 1372 cm^{-1}, and 2252 cm^{-1}) [2], which can interfere with detection. For most analytes, higher water content tends to yield stronger SERS signals. In some cases, a signal is only observed at high potentials in the presence of high acetonitrile content.

We also evaluated the effect of supporting electrolytes - considered here both for their role in signal enhancement and their compatibility with HPLC separation. Significant variations in signal intensity were observed depending on the electrolyte used. Most common inorganic salts did not damage the SERS substrate but did affect signal intensity. Compatible cations based on our experience include: NH_4^+, Na^+, K^+, Ca^{2+}, and Bu_4N^+. Compatible anions include: citrate, acetate, formiate (although it gives low intensities with crystal violet), Cl^- (short-term), NO_3^-, SO_4^{2-}, HPO_4^{2-}, and borax. Incompatible anions include: Br^-, I^-, and $S_2O_3^{2-}$.

The detection of each analyte needs to be optimized separately within the described parameters, including the individual potential program. Regarding the electrical potential program, oxidative potentials destroy the SERS substrate.

Additional solvents and HPLC conditions are currently under investigation. It is important to note that all observations reported in this chapter are based on our current experimental experience and should be interpreted with caution. Further experimental validation is required to confirm the general applicability and reproducibility of these findings, as well as the precise quantification of the ageing of the SERS substrate.

References

(1) Blaha, M. E.; Das, A.; Belder, D. *Analytical and bioanalytical chemistry* **2025**, DOI: 10.1007/s00216-025-05763-w.
(2) Salinas-Luna, J.; Mentado-Morales, J. *Phys. Scr.* **2024**, DOI: 10.1088/1402-4896/ad1025.
